# Increased optic atrophy type 1 expression protects retinal ganglion cells in a mouse model of glaucoma

**Published:** 2010-07-15

**Authors:** Won-Kyu Ju, Keun-Young Kim, Karen X. Duong-Polk, James D. Lindsey, Mark H. Ellisman, Robert N. Weinreb

**Affiliations:** 1 The Sophie and Arthur Brody Optic Nerve Laboratory, Hamilton Glaucoma Center, University of California San Diego, La Jolla, CA; 2 Center for Research on Biological Systems, National Center for Microscopy and Imaging Research and Department of Neuroscience, University of California San Diego School of Medicine, La Jolla, CA

## Abstract

**Purpose:**

The goal of this study is to determine whether increased optic atrophy type 1 (OPA1) expression protects against retinal ganglion cell (RGC) death in glaucomatous DBA/2J mice.

**Methods:**

Intraocular pressure in DBA/2J mice was measured, and pre-glaucomatous DBA/2J mice eyes were transfected with recombinant adeno-associated virus serotype 2 (AAV2) constructs including AAV2-wild type (WT) mOPA1 for two months. Increased OPA1 expression was confirmed by western blotting and RGC survival was assessed by retrograde labeling with FluoroGold. In addition, apoptotic cell death and mitochondrial structure were determined in AAV2-WT mOPA1-transfected differentiated RGC-5 cells exposed to elevated hydrostatic pressure (30 mmHg) for three days.

**Results:**

WT AAV2-mOPA1 transfection significantly increased 90 kDa and 80 kDa OPA1 isoforms in the retina of glaucomatous DBA/2J mice. OPA1 immunoreactivity was increased in the inner nuclear layer, inner plexiform layer, and ganglion cell layer in nine month-old glaucomatous DBA/2J mice transfected with AAV2-WT mOPA1. Overexpression of OPA1 significantly increased RGC survival at two months after AAV2-WT mOPA1 transfection, and decreased activation of both astroglia and microglia in the retina of glaucomatous DBA/2J mice. Also, overexpression of OPA1 in differentiated RGC-5 cells resulted in less apoptotic cell death and blocked mitochondrial fission following elevated hydrostatic pressure.

**Conclusions:**

OPA1 can directly modulate RGC survival, and increasing OPA1 expression may protect against RGC death in glaucomatous optic neuropathy.

## Introduction

Elevated intraocular pressure (IOP) is an important risk factor for optic nerve head damage and retinal ganglion cell (RGC) death in glaucoma [1]. However, the precise pathophysiological relationship between elevated IOP and RGC death remains poorly understood. Mitochondrial fission-mediated mitochondrial dysfunction has been linked to neuronal cell death in both acute and chronic neurodegenerative disorders [2-6]. Further, it has been hypothesized that alterations of optic atrophy type 1 (OPA1), a mitochondrial fusion protein, contribute to RGC death in glaucoma [2,3]. However, the direct relationships among elevated IOP, OPA1 expression, and mitochondrial dysfunction-mediated RGC death in glaucoma remain unknown.

Mitochondria are autonomous and morphologically dynamic organelles that structurally reflect a precise balance of ongoing fission and fusion within a healthy cell [4-8]. This balance is regulated by a family of dynamin-related GTPases that exert opposing effects. OPA1—the human ortholog of Mgm1p/Msp1p—and mitofusins are required for mitochondria fusion. In contrast, dynamin-related protein1 promotes mitochondrial fission [5,9]. Mutations in OPA1, which is involved in various processes related to mitochondrial inner membrane structural dynamics, are linked with neurodegenerative disease in humans and cause autosomal dominant optic atrophy, a common form of hereditary optic neuropathy [10,11].

OPA1 is expressed in the soma and axons of the RGCs as well as in the horizontal cells [12-15]. Emerging evidence suggests that downregulation of OPA1 causes mitochondrial fission leading to cytochrome c release and apoptosis in HeLa cells, as well as induces aggregation of the mitochondrial network in purified RGCs [16-19]. In contrast, increased OPA1 expression protects cells from apoptosis by preventing cytochrome c release and by stabilizing the shape of mitochondrial cristae [20,21]. Together, these results suggest that the direct modulation of OPA1 could regulate RGC survival.

Hence, the present study was undertaken to determine whether increasing OPA1 expression protects against RGC death in glaucomatous DBA/2J mice or in differentiated RGC-5 cells exposed to elevated hydrostatic pressure.

## Methods

### Plasmid and recombinant adeno-associated virus serotype 2 constructs

The wild type (WT) mOPA1 plasmid [22] was provided by Drs. Takumi Misaka (University of Tokyo, Japan) and Yoshihiro Kubo (National Institute for Physiologic Sciences, Japan). It is used to produce recombinant adeno-associated virus serotype 2 (AAV2)-WT mOPA1 (4.1×10^11^ GC/ml) using the pAAV-CMV-shuttle by Applied Viromics (Fremont, CA). AAV2-CMV-GFP (1×10^12^ GC/ml) and AAV2 Null (1×10^12^ GC/ml) were purchased from Applied Viromics. AAV2 Null that does not contain any gene insert is made using pAAV-CMV-shuttle.

### Animals

All procedures concerning animals were in accordance with the ARVO Statement for the Use of Animals in Ophthalmic Vision Research and under the protocols approved by institutional IACUC committees at the University of California-San Diego. Adult female DBA/2J (The Jackson Laboratory, Bar Harbor, ME) and C57BL/6 (Harlan Laboratories, Indianapolis, IN) mice were housed in covered cages, fed with a standard rodent diet ad libitum, and kept on a12 h:12 h light-dark cycle. Prior studies have shown that IOP and optic nerves appear to be normal in three-month-old DBA/2J mice [23-26]. IOP elevation onset typically occurs between five and seven months of age, and by nine months of age, IOP-associated optic nerve axon loss is well advanced [23-27].

### IOP measurement

IOP measurement was performed as described previously [2,23,28]. Starting at six month of age, IOP was measured monthly. Inclusion criteria for this study was IOP less than 20 mmHg at six months of age and IOP greater than 20 mmHg at nine months of age (n=50 mice). Briefly, mice were anesthetized with a mixture of ketamine (100 mg/kg, Ketaset; Fort Dodge Animal Health, Fort Dodge, IA) and xylazine (9 mg/kg, TranquiVed; Vedeco, Inc., St. Joseph, MO), and then pressure was measured by the insertion of a sterilized, water-filled microneedle with an external diameter of 50 to 70 μm connected to a pressure transducer into the anterior chamber.

### Intravitreal injection of adeno-associated virus serotype 2 constructs

The seven-month-old DBA/2J and C57BL/6 mice were anesthetized with a mixture of ketamine and xylazine, and with topical 1% proparacaine eye drops. To facilitate the injection, a hole was made in the sclera 3 mm posterior to the superotemporal limbus. A Hamilton syringe with 34-gauge needle (Hamilton Co., Reno, NV) was used to inject 5 µl of AAV2 Null, AAV2-CMV-GFP, or AAV2-WT mOPA1 into the vitreous humor. Injections were given slowly over 1 min and the needle was maintained in position for an additional 10 min to minimize vector loss through the injection tract. At nine months of age, the mice were killed by an intraperitoneal (IP) injection of a mixture ketamine and xylazine, and retinal tissues were prepared as below.

### Retrograde labeling of retinal ganglion cells

One week before sacrifice, retrograde tracers were microinjected bilaterally into the superior colliculi of mice anesthetized with a mixture of ketamine and xylazine in a stereotactic apparatus, as previously described [14]. 1,1’-dioctadecyl-3,3,3′,3′-tetramethylindocarbocyanine perchlorate (DiI; 1 µl/injection of 5%; diluted in 100% ethanol; Molecular Probes) was used for double labeling with AAV2-CMV-GFP, or FluoroGold (1 µl/injection of 4%; diluted in saline; Fluorochrome Inc., Englewood, CO) was used for RGC counting. RGC counting was done by two investigators in a masked fashion and the scores were averaged.

### Tissue preparations

The mice were anesthetized with isoflurane and killed by an IP injection of a mixture of ketamine and xylazine. For immunohistochemistry, the retinas were dissected from the choroid and fixed with 4% paraformaldehyde in phosphate buffered saline (PBS, pH 7.4) for 2 h at 4 °C. After several washes in PBS, retinas were dehydrated through graded ethanols and then embedded in a polyester wax as described previously [14]. For the western blot analyses, whole retinas were immediately used.

### Western blot analysis

Retinas were dissected from the sclera and then immediately homogenized in a glass-Teflon Potter homogenizer in a lysis buffer (20 mM Hepes, pH 7.5/10 mM KCl/1.5 mM MgCl_2_/1 mM EDTA/1 mM EGTA/1 mM DTT/0.5% CHAPS/complete protease inhibitors; Roche Biochemicals, Indianapolis, IN). Ten micrograms of pooled retinas (n=4 retinas/group) from each group were separated by PAGE and electro-transferred to PVDF membranes. The membrane was blocked with 5% nonfat dry milk/0.05% Tween-20/PBS; incubated with mouse monoclonal anti-OPA1 antibody (H-300, 1:1,000; BD Transduction Laboratories, San Diego, CA), mouse monoclonal anti-glial fibrillary acidic protein antibody (GFAP, 1:3000; Sigma, St. Louis, MO), or mouse monoclonal anti-actin antibody (1:2,000; Chemicon-Millipore, Billerica, MA); rinsed with 0.05% Tween-20/PBS; incubated with peroxidase-conjugated goat antimouse IgG (1:2,000; Bio-Rad, Hercules, CA); and developed using chemiluminescence detection (ECL Plus, GE Healthcare Bio-Sciences, Piscataway, NJ). Images were analyzed with a digital fluorescence imager (Storm 860; GE Healthcare Bio-Sciences) and band densities were normalized using actin as the calibrator with ImageQuant TL (GE Healthcare Bio-Sciences).

### Immunohistochemical analyses

Immunohistochemical staining of 7-µm retinal sections or wholemounts was done by the immunofluorescent method. Primary antibodies were mouse monoclonal anti-neurofilament (1:100; Sigma) to identify axons, mouse monoclonal anti-GFAP (1:300; Sigma) to identify astrocytes, rabbit polyclonal anti-Iba 1 (1:100; Wako Chemicals, Osaka, Japan) to identify microglial cells, and rabbit polyclonal anti-mOPA1 (1:500, a gift from Takumi Misaka, University of Tokyo, and Yoshihiro Kubo, National Institute for Physiologic Sciences, Japan) [22]. To prevent a non-specific background, tissues were incubated with 3% normal donkey serum/PBS for 1 h at room temperature and then with the primary antibodies for 24 or 72 h at 4 °C. After several wash steps, the tissues were incubated with the secondary antibodies, Alexa Fluor 488 dye-conjugated goat antimouse IgG (1:100; Invitrogen, Carlsbad, CA), Alexa Fluor 488 dye-conjugated goat antirabbit IgG (1:100; Invitrogen), or Alexa Fluor 569 dye-conjugated goat antirabbit IgG (1:100; Invitrogen), for 4 or 24 h at 4 °C and then washed with PBS. The sections were counterstained with the nucleic acid stain Hoechst 33342 (1 µg/ml, Invitrogen) in PBS. Images were acquired with confocal microscopy (Olympus FluoView1000; Olympus, Tokyo, Japan). Image exposures were the same for all tissue sections and were stored on a computer (Photoshop files; Adobe Systems Inc., San Jose, CA).

### Retinal ganglion cell-5 cell studies

RGC-5 cells were grown on 12 mm coverslips in 24 well plates as previously described [3,29-32] and cotransfected by exposure to a medium containing AAV2 Null (1×10^12^ GC/ml) or AAV2-WT mOPA1 (4.1×10^11^ GC/ml). Differentiation of RGC-5 cells was induced three days after treatment with succinyl concanavalin A (50 µg/ml, Sigma, St. Louis, MO). A pressurized incubator was used to expose the cells to elevated hydrostatic pressure as previously described [3,30]. To determine mitochondrial morphology and cell death, nuclei were stained with Hoechst 33342 (1 μg/ml; Molecular Probes; 10 min at room temperature) and visualized using fluorescence microscopy. Each evaluated cell was scored as having normal or pyknotic (apoptotic) nuclei in several fields. At least 200 cells were scored for each experimental condition. Results were expressed as a percentage of cells with apoptotic nuclei among the total cells that were counted by two investigators in a masked fashion. Mitochondria were labeled by the addition of red fluorescent mitochondrial dye to the cultures (100 nM final concentration; MitoTracker Red CMXRos; Invitrogen) as described previously [14].

### Statistical analysis

The data were presented as the mean±SD. Comparisons of two or three experimental conditions were evaluated using the paired or unpaired Student *t* test, or one-way ANOVA and Bonferroni *t* test. p<0.05 was considered to be statistically significant.

## Results

### Effect of increased optic atrophy type 1 expression on retinal ganglion cell survival

**Figure 1:**
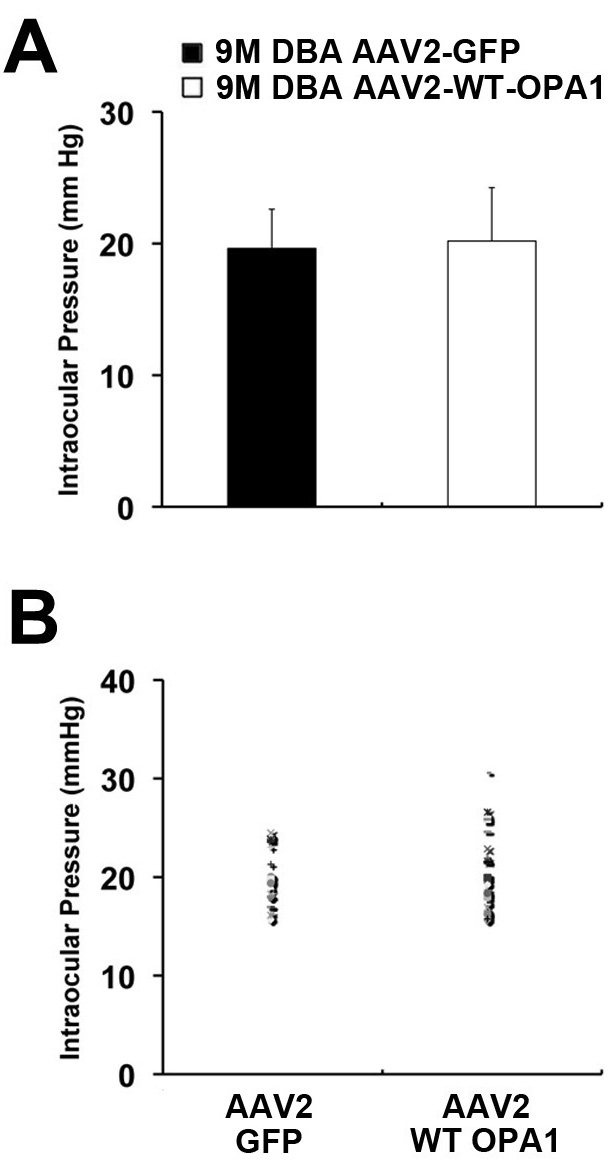
Intraocular pressure (IOP) measurement in nine-month-old glaucomatous DBA/2J mice following transfection with adeno-associated virus serotype 2-cytomegalovirus-green fluorescent protein (AAV2-CMV-GFP) or adeno-associated virus serotype 2-wild type (AAV2-WT) optic atrophy type 1 (mOPA1). **A**: Similar mean IOP was observed in the eyes of nine-month-old DBA/2J mice injected with either AAV2-CMV-GFP (n=19) or AAV2-WT mOPA1 (n=29). **B**: Distribution of IOP in the eyes of mice nine months of age following injection of vectors. Data represent the mean±SD. There was no significant IOP difference.

Mean IOP was 19.6±2.9 mmHg in the eyes of the nine-month-old DBA/2J mice injected with AAV2-CMV-GFP (n=19) and 20.1±4.0 mmHg in the eyes of the nine-month-old DBA/2J mice injected with AAV2-WT mOPA1 (n=29; Figure 1). These were no significant differences in IOP between the AAV2-CMV-GFP and AAV2-WT mOPA1-injected glaucomatous DBA/2J mice at nine months of age.

**Figure 2:**
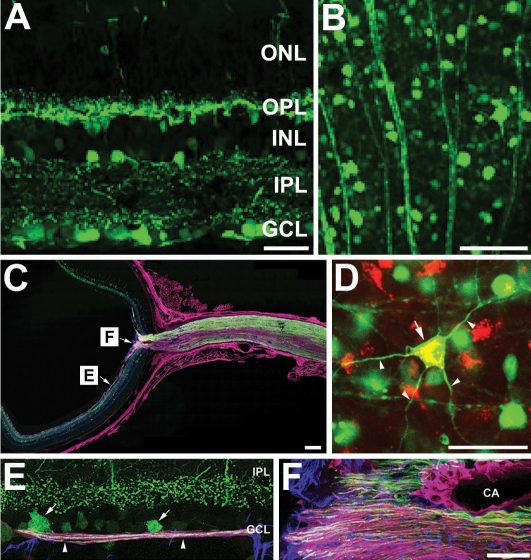
Adeno-associated virus serotype 2-cytomegalovirus-green fluorescent protein (AAV2-CMV-GFP) labeling in the retina and optic nerve of six-month-old DBA/2J mice. **A**: Normal appearance of retinal cross section. **B**: The ganglion cell layer (GCL) from the retinal flatmount. **C**: Optic nerve fiber staining in a whole eye section after triple labeling with AAV2-CMV-GFP (green), neurofilament (red), and glial fibrillary acidic protein (GFAP; blue). **D**: The entire dendritic tree (arrowheads) and cell body (arrow) of retinal ganglion cell (RGC) labeled with AAV2-CMV-GFP (green) and DiI (red). **E**: Enlargement of the retinal section shown in panel **C** to illustrate staining in the RGC somas (arrows) and axons (arrowheads) in the GCL. **F**: Enlargement of the optic nerve head shown in panel **C** demonstrates triple labeling with AAV2-CMV-GFP (green), neurofilament (red), and GFAP (blue). Abbreviations: IPL represents inner plexiform layer; GCL represents ganglion cell layer; CA represents central artery. Scale bar indicates 20 µm (**A**, **D**-**F**), 50 µm (**B**), and 100 µm (**C**).

To evaluate the targeting of recombinant AAV2 constructs, the intravitreal injection of AAV2-CMV-GFP was combined with retrograde labeling of RGCs by a DiI injection into the superior colliculi. Following the AAV2-CMV-GFP injection in the seven month-old DBA/2J mice, GFP expression was observed throughout the whole retina and optic nerve in the DBA/2J mice at nine months of age (Figure 2A-F). Double labeling with DiI showed the entire dendritic tree of a subset of RGCs labeled with GFP (Figure 2D). Specifically, GFP was filled in the RGC somas and their axons in the ganglion cell layer (GCL) and nerve fiber layer (Figure 2E), and in the optic nerve head (Figure 2F).

**Figure 3:**
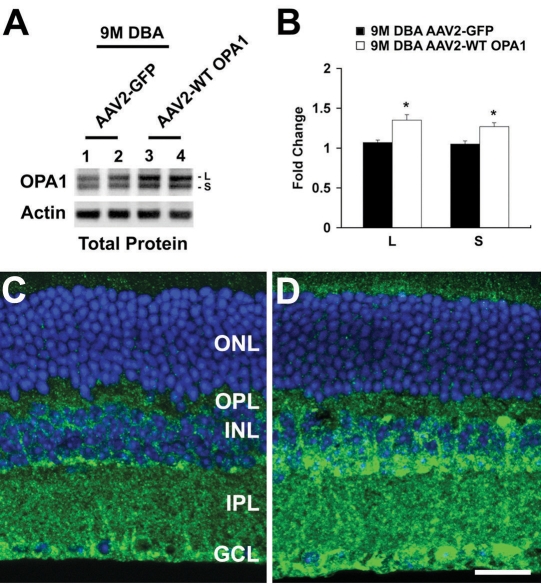
Retinal ganglion cell (RGC) survival in glaucomatous DBA/2J mice following transfection with adeno-associated virus serotype 2-cytomegalovirus-green fluorescent protein (AAV2-CMV-GFP) or adeno-associated virus serotype 2-wild type (AAV2-WT) optic atrophy type 1 (mOPA1). **A**, **B**: The OPA1 antibody recognized 90-kDa (L) and 80-kDa (S) isoforms of OPA1 protein in the total retinal protein extracts of glaucomatous DBA/2J mice transfected with AAV2-CMV-GFP. Conversely, AAV2-WT mOPA1-trasfected glaucomatous mice had significantly increased isoforms of the OPA1 protein compared with glaucomatous DBA/2J mice treated with AAV2-CMV-GFP (n=4 retinas/group). Data represent the mean±SD *Significant at p<0.05 compared with nine-month-old glaucomatous DBA/2J mice transfected with AAV2 Null. **C**: OPA1 immunoreactivity in glaucomatous DBA/2J mice transfected with AAV2 Null. **D**: OPA1 immunoreactivity in glaucomatous DBA/2J mice transfected with AAV2-CMV-WT mOPA1. Abbreviations: ONL represents outer nuclear layer; OPL represents outer nuclear layer; IPL represents inner plexiform layer; GCL represents ganglion cell layer; CA represents central artery. Scale bar indicates 20 µm (**C**, **D**).

**Figure 4:**
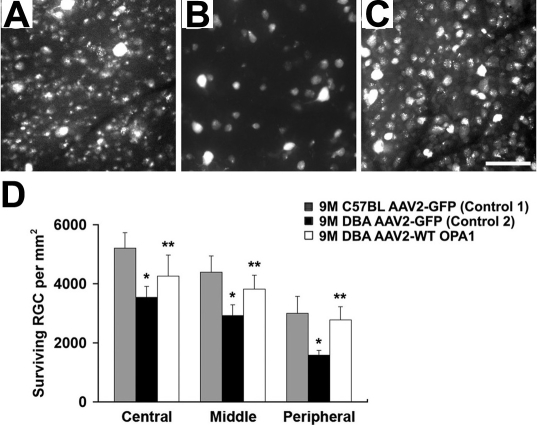
Retinal ganglion cell (RGC) survival in nine-month-old glaucomatous DBA/2J mice following transfection with adeno-associated virus serotype 2-cytomegalovirus-green fluorescent protein (AAV2-CMV-GFP) or adeno-associated virus serotype 2-wild type (AAV2-WT) optic atrophy type 1 (mOPA1). The representative flatmount photomicrographs are from the middle retina following retrograde labeling with FluoroGold. **A**: Non-glaucomatous C57BL/6 mice transfected with AAV2-CMV-GFP; n=4 retinal flatmounts/mice/group. **B**: Glaucomatous DBA/2J mice transfected with AAV2-CMV-GFP; n=4 retinal flatmounts/mice/group. **C**: Glaucomatous DBA/2J mice transfected with AAV2-WT mOPA1; n=7 retinal flatmounts/mice/group. **D**: The quantitative analysis of RGC survival. Data represent the mean±SD *Significant at p<0.05 compared with non-glaucomatous C57BL/6 mice transfected with AAV2-CMV-GFP or **Significant at p<0.05 compared with glaucomatous DBA/2J mice transfected with AAV2-CMV-GFP. Scale bar indicates 50 µm (**C**-**E**).

The OPA1 antibody recognized two major isoforms of the OPA1 protein (a 90-kDa [called the large or L isoform] and an 80-kDa [called small or S isoform]) in the total retinal protein extracts of the nine-month-old glaucomatous DBA/2J mice transfected with AAV2-CMV-GFP. In contrast, both the L and S isoforms of the OPA1 protein were significantly increased in the nine-month-old glaucomatous DBA/2J mice transfected with AAV2-WT mOPA1 (n=4 retinas/group, p<0.05, Figure 3A,B). Further, OPA1 immunoreactivity was increased in the inner nuclear layer, inner plexiform layer, and ganglion cell layer in the nine-month-old glaucomatous DBA/2J mice transfected with AAV2-WT mOPA1 compared with the nine-month-old glaucomatous DBA/2J mice transfected with AAV2 Null (Figure 3C). In comparison with the nine-month-old non-glaucomatous C57BL/6 mice transfected with AAV2-CMV-GFP (n=4 retinas, Figure 4A, Table 1), the nine-month-old glaucomatous DBA/2J mice transfected with AAV2-CMV-GFP had 38% less RGCs in the central, middle, and peripheral retinas combined (n=5 retinas, Figure 4B,D, Table 1; p<0.05). The nine-month-old glaucomatous DBA/2J mice transfected with AAV2-WT mOPA1 had 13% less RGCs than the nine-month-old non-glaucomatous C57BL/6 mice transfected with AAV2-CMV-GFP (n=7 retinas, Figure 4C,D, Table 1; p<0.05).

**Table 1:** Effects of increased optic atrophy type 1 (OPA1) expression on the central, middle and peripheral retinal ganglion cell (RGC) survival from 9-month-old glaucomatous DBA/2J mice.

**Strain**	**Age (mo)**	**Treatment**	**RGC density per retina (RGCs/mm^2^)**		
			**Central**	**Middle**	**Peripheral**
C57BL/6	9	AAV2-CMV-GFP	5208±522	4389±555	3000±570
DBA/2J	9	AAV2-CMV-GFP	3539±370*	2922±367*	1585±160*
DBA/2J	9	AAV2-WT mOPA1	4260±712**	3820±470**	2775±450**

Values are given as means±SD. Comparison of three experimental conditions was evaluated using one-way ANOVA (ANOVA) and the Bonferroni *t*-test. *Significant at p<0.05 compared with non-glaucomatous C57BL/6 mice transfected with adeno-associated virus serotype 2-cytomegalovirus-green fluorescent protein (AAV2-CMV-GFP), n=4 retinal flatmounts/mice/group. **Significant at p<0.05 compared with glaucomatous DBA/2J mice transfected with AAV2-CMV-GFP, n=7 retinal flatmounts/mice/group.

### Effect of increased optic atrophy type 1 expression on retinal astroglial and microglial activation

**Figure 5:**
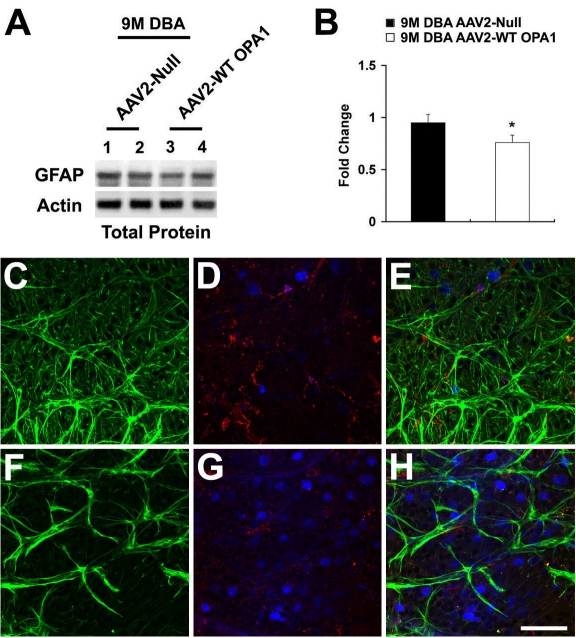
Increased optic atrophy type 1 (OPA1) expression inhibits activation of astroglia and microglia in the retinas of glaucomatous DBA/2J mice. **A, B**: Adeno-associated virus serotype 2-wild type (AAV2-WT) mOPA1-trasfected glaucomatous mice significantly decreased GFAP protein compared with glaucomatous DBA/2J mice treated with AAV2 Null. Relative intensity of chemiluminescence for each protein band was normalized with actin used as the calibrator; n=4 retinas/group. Data represent the mean±SD *Significant at p<0.05 compared with glaucomatous DBA/2J mice transfected with AAV2-CMV Null. **C, D**: Glaucomatous retina transfected with AAV2 Null exhibited significantly induced activations of both astroglia (green, **C**) and microglia (red, **D**). **E**: Merged retina with triple labeling. **F, G**: Glaucomatous retina transfected with AAV2-WT mOPA1 significantly reduced activation of both astroglia (green, **F**) and microglia (red, **G**). **H**: Merged retina with triple labeling. The blue color shows FluroGold-labeled RGCs. Scale bar indicates 50 µm (**C-H**).

In comparison with the nine-month-old glaucomatous DBA/2J mice transfected with AAV2 Null, GFAP expression was significantly decreased in the nine-month-old glaucomatous DBA/2J mice transfected with AAV2-WT mOPA1 (Figure 5A,B; p<0.05). In parallel, AAV2 Null transfection increased GFAP-positive astroglial activation in the GCL of the nine-month-old glaucomatous DBA/2J mice (Figure 5C). It appeared that Iba 1-positive microglial activation was stronger than astroglial activation in the GCL of the nine-month-old glaucomatous DBA/2J mice transfected with AAV2 Null (Figure 5D). However, the nine-month-old glaucomatous DBA/2J mice transfected with AAV2-WT mOPA1 had less activation of both astroglia and microglia in the retina (Figure 5F-H). In addition, retinas from the nine-month-old glaucomatous DBA/2J mice transfected with AAV2 Null showed a similar activation by immunohistochemistry of both astroglia and microglia as the untransfected nine-month-old glaucomatous DBA/2J mice (data not shown).

### Effect of increased optic atrophy type 1 expression on pressurized retinal ganglion cell-5 cells

**Figure 6:**
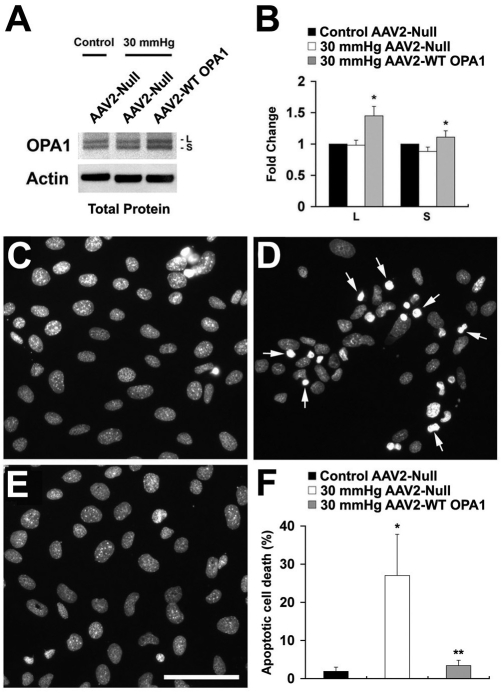
Increased optic atrophy type 1 (OPA1) expression blocks apoptotic cell death in differentiated retinal ganglion cell (RGC)-5 cells exposed to elevated hydrostatic pressure (30 mmHg) for 3 days. **A, B**: The OPA1 antibody recognized 90-kDa (L) and 80-kDa (S) isoforms of OPA1 protein in the total protein extracts of non-pressurized or pressurized cells transfected with AAV2 Null or AAV2-WT mOPA1. Compared with pressurized cells transfected with AAV2 Null, pressurized cells transfected with AAV2-WT mOPA1 significantly increased both isoforms of OPA1 protein at 3 days. Data represent the mean±SD *Significant at p<0.05 compared with pressurized cells transfected with AAV2 Null (n=4 retinas/group). **C-E**: Non-pressurized control cells transfected with AAV2 Null (**C**), pressurized cells transfected with AAV2 Null (**D**), and pressurized cells transfected with AAV2-WT mOPA1 (**E**). The cells are counterstained with Hoechst 33342. (**F**) The quantitative analysis of apoptotic cell death (n=200 cells per group, arrows). *Significant at p<0.05 compared with non-pressurized control cells transfected with AAV2 Null, **Significant at p<0.05 compared with pressurized cells transfected with AAV2 Null. Scale bar indicates 50 µm (**C-E**).

As in the protein extracts from the retina, the OPA1 antibody recognized two isoforms of the OPA1 protein (a 90-kDa [L] and an 80-kDa [S]) in the total protein extracts from RGC-5 cells (Figure 6A,B). There was no significant difference in OPA1 expression between the AAV2 Null-transfected non-pressurized control and pressurized RGC-5 cells. In contrast, both isoforms of the OPA1 protein were significantly increased in the pressurized RGC-5 cells transfected with AAV2-WT mOPA1 (Figure 6A,B, p<0.05). Hoechst 33342 staining showed that non-pressurized control RGC-5 cells transfected with AAV2 Null contained normal appearing nuclei (Figure 6C). In contrast, pressurized RGC-5 cells transfected with AAV2 Null showed a significant apoptotic nuclei (Figure 6D). However, increased OPA1 expression by transfection of AAV2-WT mOPA1 resulted in almost complete protection from apoptotic cell death in pressurized RGC-5 cells (Figure 6E,F; p<0.05).

**Figure 7:**
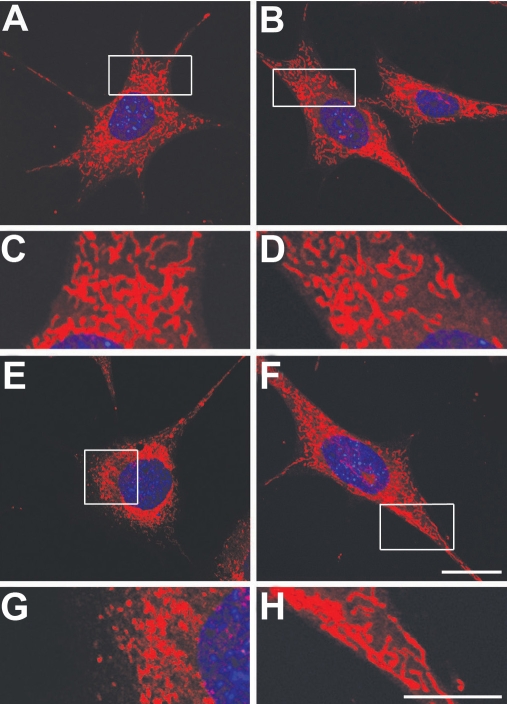
Increased optic atrophy type 1 (OPA1) expression blocks mitochondrial fission in differentiated retinal ganglion cell (RGC)-5 cells exposed to elevated hydrostatic pressure (30 mmHg) for three days. Mitochondria are stained with MitoTracker Red. **A, C**: Non-pressurized control cells without transfection. **B, D**: Non-pressurized control cells transfected with AAV2 Null. **E,G**: Pressurized cells transfected with AAV2 Null. **F,H**: Pressurized cells transfected with AAV2-WT mOPA1. High magnification showed that increased OPA1 expression blocked mitochondrial fission in pressurized cells that are counterstained with Hoechst 33342 (blue). Scale bar indicates 10 µm (**A,C, F, H**).

Mitochondria in the non-pressurized control RGC-5 cells transfected with AAV2 Null showed a typical filamentous and fused mitochondrial network at three days (Figure 7A-D). Elevated hydrostatic pressure induced mitochondrial fission—characterized by the conversion of tubular fused mitochondria into isolated small organelles—in the pressurized RGC-5 cells transfected with AAV2 Null at 3 days (Figure 7E,G). In contrast, increased OPA1 expression by transfection of AAV2-WT mOPA1 blocked mitochondrial fission in the pressurized RGC-5 cells (Figure 7F,H).

## Discussion

These results provide the first direct evidence that increased OPA1 expression using an AAV2 vector system can increase RGC survival in glaucomatous DBA/2J mice with elevated IOP in vivo and in differentiated RGC-5 cells exposed to elevated hydrostatic pressure in vitro. Moreover, increased OPA1 expression significantly reduced the activation of both astroglia and microglia in glaucomatous retina, and blocked apoptotic cell death in RGC-5 cells exposed to elevated hydrostatic pressure. These findings suggest that increasing OPA1 expression may protect RGCs against glaucomatous damage.

Emerging evidence indicates that mitochondrial structural and functional dynamics play an important role in cell and animal physiology. Imbalance in the control of mitochondrial fusion and fission dramatically alters overall mitochondrial morphology [9]. Recent evidence suggests that excessive mitochondrial fission can lead to the breakdown of the mitochondrial network, loss of mitochondrial DNA, and respiratory defects in mammalian cells [33-35]. Intriguingly, we recently reported that elevated IOP induces mitochondrial fission, decreases OPA1 gene expression, triggers OPA1 and cytochrome c release, and induces apoptotic cell death in the retinas and optic nerves of glaucomatous DBA/2J mice [2,3]. Together with the present findings, these observations indicate that reduced OPA1 may contribute to RGC pathophysiology in glaucoma.

Gene delivery strategies by recombinant AAV2 constructs have been explored as a method to prevent RGC loss in experimental animal models of glaucoma [36,37]. Because we previously found that all neurons containing OPA1 immunoreactivity were co-labeled with FluoroGold-positive RGCs in a mouse model of glaucoma [14,38], we developed a gene therapy strategy using the transfection of a recombinant AAV2-WT mOPA1 construct to investigate whether overexpression of OPA1 increases RGC survival in glaucomatous DBA/2J mice with elevated IOP. Importantly, our findings demonstrated the first evidence that increased OPA1 expression significantly blocked RGC loss in glaucomatous DBA/2J mice. In comparison between non-glaucomatous C57BL/6 mice and OPA1-expressing glaucomatous mice, we found no difference in RGC loss in the peripheral retinas of these strains (p>0.05). In the central and middle retina, however, RGC survival was 18.7% greater in the non-glaucomatous C57BL/6 mice than in the OPA1-expressing glaucomatous DBA/2J mice (p<0.05). As RGC loss was much greater in the non-transfected glaucomatous DBA/2J mice, these results indicated that our induction of OPA1 provided partial protection against RGC loss. In addition to protecting RGCs, it was confirmed that increased OPA1 expression significantly reduced the activation of both astroglia and microglia in glaucomatous retinas. Astroglial or microglial activation coincides with RGC degeneration in glaucomatous retinas in humans, rats, or mice [39-47]. Hence, the present results suggest that increased OPA1 expression in RGCs may indirectly reduce astroglial or microglial activation as a result of increased RGC survival. AAV2 vectors introduced into the central nervous tissue generally target neurons, but not other CNS cells such as astrocytes or microglia [48]. However, the possibility that increased OPA1 expression may directly contribute to the deactivation of astroglia or microglia should be further explored.

Growing evidence demonstrates that increased OPA1 expression protects cells from apoptosis by preventing cytochrome c release and by stabilizing the shape of mitochondrial cristae [20,21], while an OPA1 mutation or deficiency contributes to mitochondrial fission and apoptotic cell death in many cell types [16-18,49]. Further, downregulation of OPA1 causes aggregation of the mitochondrial network in purified RGCs [19]. Recently, we reported that elevated hydrostatic pressure triggers mitochondrial fission and OPA1 and cytochrome c release, as well as induces apoptotic cell death and causes a significant decrease of OPA1 gene expression in differentiated RGC-5 cells at three days [3]. In the present study, we observed that increased OPA1 expression prevented mitochondrial fission. Further, it significantly blocked apoptotic cell death in differentiated RGC-5 cells exposed to elevated hydrostatic pressure for three days, supporting our notion that increased OPA1 expression could block mitochondrial fission and subsequent apoptotic cell death in glaucomatous RGC degeneration. These results collectively indicate that pressure-induced OPA1 alteration may directly contribute to RGC death in glaucoma and that increased OPA1 expression may protect against direct pressure damage to RGCs.

Because most of pathogenic mutations in OPA1 are expected to produce a truncated non-functional OPA1 protein, it has been suggested that haploinsufficiency is a major pathomechanism in OPA1-associated autosomal dominant optic atrophy [50,51]. Moreover, recent studies reported that OPA1 mutant mice have a significantly lower (~50%) level of retinal OPA1 protein, which is associated with increased age-dependent loss of RGCs [52,53]. Consistent with these results, we observed that nine-month-old heterozygous OPA1^enu/+^ mice have significantly lower OPA1 protein levels and greater RGC loss than corresponding WT littermate control mice. Also, OPA1 immunoreactivity was decreased in the GCL of heterozygous OPA1^enu/+^ mice compared with WT littermate control mice (data not shown).

These results demonstrate a direct relationship between OPA1 expression in RGCs and RGC survival, as well as support the possibility that increased OPA1 expression may promote RGC survival in glaucoma. Thus, direct enhancement of OPA1 may provide a new strategy to protect RGC death in various optic neuropathies including glaucoma.
